# Time to Train: The Involvement of the Molecular Clock in Exercise Adaptation of Skeletal Muscle

**DOI:** 10.3389/fphys.2022.902031

**Published:** 2022-04-25

**Authors:** Shivani Mansingh, Christoph Handschin

**Affiliations:** Biozentrum, University of Basel, Basel, Switzerland

**Keywords:** circadian clock, exercise, skeletal muscle, bmal1, ROR alpha, REV-ERB, PGC-1 alpha

## Abstract

Circadian rhythms regulate a host of physiological processes in a time-dependent manner to maintain homeostasis in response to various environmental stimuli like day and night cycles, food intake, and physical activity. Disruptions in circadian rhythms due to genetic mutations, shift work, exposure to artificial light sources, aberrant eating habits, and abnormal sleep cycles can have dire consequences for health. Importantly, exercise training efficiently ameliorates many of these adverse effects and the role of skeletal muscle in mediating the benefits of exercise is a topic of great interest. However, the molecular and physiological interactions between the clock, skeletal muscle function and exercise are poorly understood, and are most likely a combination of molecular clock components directly acting in muscle as well as in concordance with other peripheral metabolic organ systems like the liver. This review aims to consolidate existing experimental evidence on the involvement of molecular clock factors in exercise adaptation of skeletal muscle and to highlight the existing gaps in knowledge that need to be investigated to develop therapeutic avenues for diseases that are associated with these systems.

## Introduction

Physiological and behavioral processes of almost all organisms are dependent on time-of-day. In mammals, light entrains these processes in the hypothalamic suprachiasmatic nucleus (SCN), which forms the central clock, and tightly synchronizes whole-body function to a 24-h light-dark cycle (Ko and Takahashi, 2006). Systemic cues like the ‘master clock’ in the SCN, metabolic cues like feeding, and cues such as stress or exercise influence the regulation of circadian factors to generate tissue-specific changes in transcription, mRNA accumulation and translation (Takahashi, 2017). Fluctuations of these perturbations are closely tied to day-night cycles and thus serve as time cues, so-called Zeitgeber, that modulate the clock in peripheral tissues (Takahashi, 2017). While the core clock machinery is conserved across all cell types, clock output is highly tissue-specific and dependent on the downstream targets.

**FIGURE 1:**
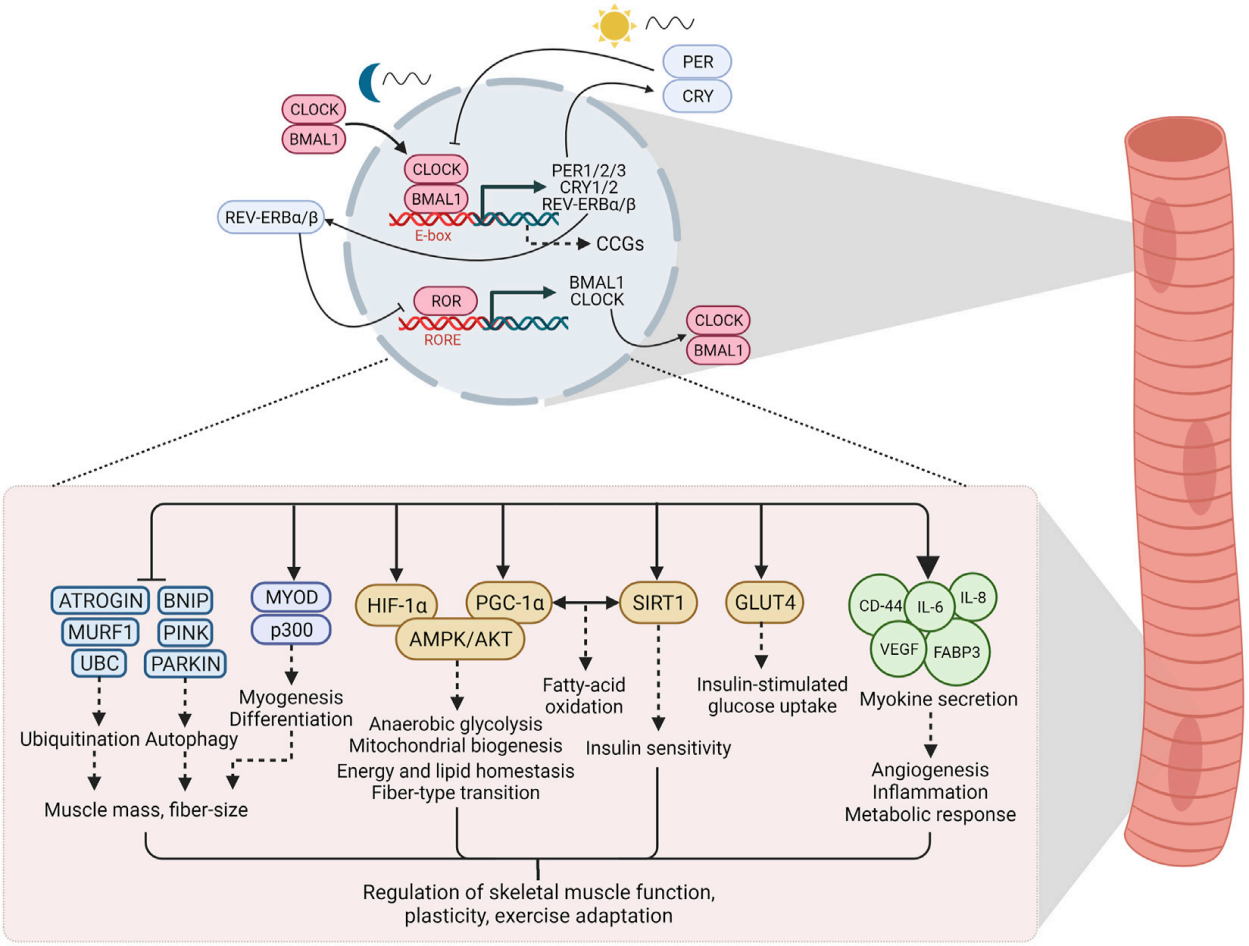
The circadian clock and downstream signaling in skeletal muscle. Clock factors regulate the expression of hundreds of genes, often termed clock-controlled genes (CCGs), outside of the circadian feedback loop. In skeletal muscle, these downstream targets are involved in a host of signaling processes that regulate skeletal muscle function, plasticity and adaptation to exercise. See text for details. Abbreviations: CLOCK, Circadian Locomotor Output Cycles Kaput; BMAL1, Brain and Muscle ARNT-Like 1; PER, Period; CRY, cryptochrome circadian regulator; ROR, retinoic acid-related orphan receptors; RORE, ROR/REV-ERB-response element; CCGs, clock controlled genes; UBC, Ubiquitin C; MURF1, Muscle RING-finger protein-1; BNIP, BCL2 Interacting Protein; MYOD, myoblast determination protein; HIF-1α, hypoxia-inducible factor 1α; PGC-1α, peroxisome proliferator-activated receptor *γ* coactivator 1α; AMPK, AMP/ADP-dependent protein kinase; AKT, Protein kinase B; SIRT1, Sirtuin 1; GLUT4, Glucose transporter type 4; CD-44, cluster of differentiation 44; IL-6, Interleukin 6; IL-8, Interleukin 8; VEGF, vascular endothelial growth factor; FAPB3, fatty acid binding protein 3. Created with BioRender.com.

The core machinery of the circadian clock in mammals consists of autoregulatory transcription and translation feedback loops that respond to environmental and metabolic stimuli to maintain temporal homeostasis (Ko and Takahashi, 2006; Takahashi, 2017). The primary arm of the core loop consists of Circadian Locomotor Output Cycles Kaput (CLOCK) and Brain and Muscle ARNT-Like 1 (BMAL1), members of the basic-helix-loop-helix/Per-ARNT-SIM (bHLH-PAS) family of transcriptional regulators (Ko and Takahashi, 2006; Partch et al., 2014; Takahashi, 2017). These proteins reach their peak activity during the inactive phase (humans: night, mice: day) and heterodimerize to bind to E-box elements in the nucleus, thereby driving the transcription of the target genes Period (PER1/2/3) and cryptochrome circadian regulator (CRY1/2) (Partch et al., 2014). PER and CRY exhibit peak activity at the beginning of the active phase and form repressor complexes that inhibit transcriptional activity of CLOCK and BMAL1, thus including their own gene expression (Shearman et al., 2000; Partch et al., 2014; Ye et al., 2014). These feedback cycles last approximately 24 h (“*circa diem*”) and define the period length. The secondary arm of the loop is comprised of the retinoic acid-related orphan receptors (RORα/β/γ, *NR1F1/2/3*) and REV-ERBα/β (*NR1D1/2*), which exert opposite effects on the molecular clock by activating or repressing BMAL1 transcription, respectively, *via* ROR-response elements (RORE) in the BMAL1 promoter (Ko and Takahashi, 2006; Partch et al., 2014; Takahashi, 2017). Apart from their role in maintaining clock oscillation, these factors regulate the expression of hundreds of genes outside of the circadian feedback loop. The downstream targets, clock-controlled genes (CCGs), are highly tissue-specific and govern the specific physiological consequences of the clock (Figure 1) (Takahashi, 2017).

**FIGURE 2:**
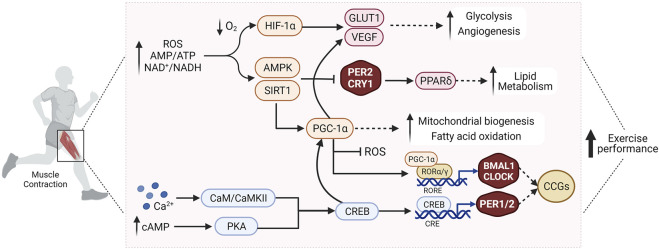
Exercise affects the molecular clock in skeletal muscle through modulation of various interconnected signaling pathways that regulate muscle metabolism and function, and improve exercise performance. See text for details. Abbreviations: ROS, reactive oxygen species; AMP, adenosine monophosphate; ATP, adenosine triphosphate; NAD^+^, nicotinamide adenine dinucleotide (oxidized form); NADH, nicotinamide adenine dinucleotide (reduced form); O_2_, oxygen; Ca^2+^, calcium; cAMP, cyclic adenosine monophosphate; HIF-1α, hypoxia-inducible factor 1α; PGC-1α, peroxisome proliferator-activated receptor *γ* coactivator 1α; AMPK, AMP/ADP-dependent protein kinase; PPARδ, peroxisome proliferator-activated receptor *δ*; SIRT1, Sirtuin 1; GLUT1, Glucose transporter type 1; VEGF, vascular endothelial growth factor; CaM/CaMKII, calmodulin/Ca2+/calmodulin-dependent protein kinase II; PKA, protein kinase A; CREB, cAMP response element-binding protein; CLOCK, Circadian Locomotor Output Cycles Kaput; BMAL1, Brain and Muscle ARNT-Like 1; PER2, Period 2; CRY1, cryptochrome circadian regulator 1; RORα/γ, retinoic acid-related orphan receptor α/γ; RORE, ROR/REV-ERB-response element; CCGs, clock controlled genes. Created with BioRender.com.

Desynchronization of circadian rhythms, e.g., due to irregular work schedules or lifestyle choices, e.g., inactivity or diet, has contributed to the increased occurrence of metabolic disorders such as obesity and insulin resistance (Potter et al., 2016). The skeletal muscle clock has emerged as an interesting candidate in understanding how clocks and tissue (patho-) physiology are linked (Turcotte and Fisher, 2008; Gabriel and Zierath, 2019). The skeletal muscle circadian transcriptome affects numerous processes, for example, proteostasis or lipid metabolism (McCarthy et al., 2007; Dyar et al., 2018), thus playing a central role in the regulation of muscle function. Similar to other cell types, the muscle clock plays an important role in anticipating metabolic fluctuations, e.g., due to feeding time or activity patterns, and therefore prepares the transition from fasting/rest phases to feeding/active phases by regulating a pleiotropic response (Dyar et al., 2014; Hodge et al., 2015; Harfmann et al., 2016). Disruption of the molecular clock in muscle exacerbates muscle atrophy and metabolic dysfunction (Zhang et al., 2020). Therefore, proper regulation of the muscle clock is essential for maintaining physiological homeostasis (Figure 1), with exercise as a potential modulator of this process (Figure 2).

## Muscle Clock Factors

### Brain and Muscle ARNT-Like 1 and Circadian Locomotor Output Cycles Kaput

BMAL1 global knockout (KO) models display muscle wasting, neurodegeneration, premature ageing, altered glucose uptake and metabolism, and are more susceptible to chronic diseases (Schiaffino et al., 2016). These phenotypes are linked to an increase in pro-inflammatory cytokines and oxidative stress due to CLOCK-mediated upregulation of nuclear factor-κB (NF-κB) in the absence of BMAL1 (Spengler et al., 2012). The defects in muscle function and structure, and accelerated aging in the BMAL1 KO, were also attributed to loss of MyoD activity (Andrews et al., 2010). Muscle-specific restoration of BMAL1 in global KO mice rescues body weight, sarcopenic pathogenesis, activity levels, and improves life span (McDearmon et al., 2006). Experiments in primary human skeletal myotubes not only confirmed the presence of cell-autonomous circadian rhythms, but also revealed time-of-day-dependent secretion of various myokines (Perrin et al., 2015). Altered secretion of myokines by the muscle transmitting signals to other tissues could thus also contribute to the rescue effect, suggesting a molecular link between the muscle clock and systemic physiology. Moreover, BMAL1 links the circadian network to mammalian target of rapamycin (mTOR) signaling by promoting protein synthesis in the muscle in response to phosphorylation by the mTOR-effector kinase S6K1, potentially linked to the muscle wasting phenotype (Lipton et al., 2015).

Similar to BMAL1, CLOCK-regulated pathways in human skeletal muscle are involved in glucose uptake, myokine secretion, and lipid metabolism (Perrin et al., 2018). Disrupting the circadian clock in muscle cells by silencing CLOCK strongly downregulated the secretion of interleukin-6 (IL-6) amongst other myokines, which is in-line with the proposed role of the muscle clock in regulating inflammation and metabolism (Perrin et al., 2015). Muscle adaptation to hypoxia is also dependent on interactions between hypoxia-inducible factor 1α (HIF-1α) and clock pathways (Peek et al., 2017). Disruption of BMAL1 in myotubes reduced levels of HIF-1α and its target genes, resulting in impaired anaerobic glycolysis and mitochondrial respiration, indicating clock-controlled fuel selection in skeletal muscle (Peek et al., 2017). Additionally, the peroxisome proliferator-activated receptor *γ* coactivator 1α (PGC-1α), a key regulator of muscle plasticity in endurance exercise, can stimulate the expression of BMAL1 by co-activating RORs, thereby potentially coupling contractile activity and energy metabolism to circadian oscillations (Lin et al., 2002; Liu et al., 2007; Summermatter et al., 2012). The skeletal muscles of Clock- and Bmal1-defective mice also have profound mitochondrial pathologies associated with altered expression of PGC-1α*,* PGC-1β and the mitochondrial transcription factor A (TFAM), which intriguingly were rescued with endurance training (Andrews et al., 2010; Pastore and Hood, 2013).

Muscle-specific inhibition of BMAL1 causes impaired insulin-stimulated glucose uptake in the muscle due to reduced levels of the insulin-dependent glucose transporter 4 (GLUT4) and regulators of translocation of this transporter (Dyar et al., 2014; Harfmann et al., 2016). CLOCK and BMAL1 also regulate insulin sensitivity in muscle myotubes through sirtuin1 (SIRT1), a NAD^+^-dependent deacetylase (Liu et al., 2016). Furthermore, BMAL1 KO mice display a shift from fast glycolytic to slow oxidative fiber-type gene expression, consistent with decreased and elevated expression of genes involved in glucose utilization and lipid utilization, respectively (Hodge et al., 2015). While some studies reported increased muscle mass and protein turnover with no differences in endurance capacity in muscle-specific BMAL1 knockouts (Dyar et al., 2018), others observed higher energy expenditure and endurance capacity associated with reduced obesity and improved metabolic profiles (Wada et al., 2018). Therefore, BMAL1/CLOCK regulate circadian gene expression, contribute to maintaining muscle oxidative capacity and coordinate time-of-day dependent shifts in carbohydrate/lipid metabolism in the muscle.

### Period and Cryptochrome Circadian Regulator

The stability and accumulation of PER and CRY proteins is tightly regulated by E3 ligases (FBXL3) and kinases (CK1ε) (Gallego and Virshup, 2007; Yoo et al., 2013). SIRT1 provides a functional link between energy homeostasis and circadian control by deacetylating PER2, thereby facilitating degradation (Asher et al., 2008). The efficacy of CRY in interacting with and silencing CLOCK-BMAL1 is also reduced by SIRT1-mediated deacetylation of BMAL1, highlighting the complex multi-level regulation of the molecular clock (Nakahata et al., 2008).

The rhythmicity of locomotor activity, measured by spontaneous wheel-running activity in mice, is disrupted in PER1/PER2 KOs, and CRY1/CRY2 KOs (van der Horst et al., 1999; Zheng et al., 2001). This might be a result of proteostatic stress in the muscle, implied by increased heat shock protein 90 (HSP90) protein levels, and a greater dependence on glycolysis (Bae et al., 2006). PER2 also binds to tuberous sclerosis complex (TSC1-TSC2), RAPTOR, and mTOR, thereby inhibiting mTOR complex 1 (mTORC1)-mediated protein synthesis (Wu et al., 2019). In line, PER2 is upregulated in mouse models of spinal muscular atrophy and denervation-induced muscle atrophy (Nakao et al., 2015; Walter et al., 2018) whereas siRNA-mediated downregulation of PER2 enhances protein synthesis and suppresses autophagy in skeletal muscle (Kalfalah et al., 2016). The knockout of *CRY1/CRY2* genes, on the other hand, enhances autophagy due to a dysregulated accumulation of PER2 and the ensuing inhibition of mTORC1 (Kalfalah et al., 2016). Furthermore, PER1/PER2 influence skeletal muscle regeneration in a circadian manner by activating insulin-like growth factor 2 (IGF2), which then engages the RAS/mitogen-activated protein kinase (MAPK) and phosphoinositide 3-kinase (PI3K)–AKT pathways (Katoku-Kikyo et al., 2021). These observations allude to circadian control of muscle function and regeneration.

CRY1/CRY2 modulate metabolic flexibility in response to circadian fluctuations by repressing the activity of peroxisome proliferator-activated receptor *δ* (PPARδ), an important regulator of energy homeostasis and lipid oxidation in the muscle, especially in the context of exercise (Jordan et al., 2017). Inhibition of CRY1/CRY2 leads to de-repression of muscle PPARδ, increased fatty acid oxidization and energy uncoupling, and ultimately enhanced endurance capacity (Jordan et al., 2017). In turn, exercise also regulates the muscle clock by activating the AMP-dependent protein kinase (AMPK). AMPK-mediated phosphorylation results in degradation of PER2 and CRY1, thereby activating PPARδ and shifting substrate utilization towards lipids during exercise to enhance performance (Lamia et al., 2009). The daytime variance in exercise capacity in mice relied on PER1/2 expression and AMPK activation (Ezagouri et al., 2019). The complex interaction between physical activity and the molecular clock is further highlighted by the induction of cAMP response element-binding protein (CREB)-mediated expression and phase-shift of PER2 in contracting muscle cells, juxtaposed to the inhibitory effect of AMPK on PER2 protein levels in this context (Small et al., 2020).

### Retinoic Acid-Related Orphan Receptors and REV-ERB

RORs and REV-ERBs activate and repress transcription of BMAL1, CLOCK, and other oscillating gene networks involved in glucose and lipid metabolism, respectively, thereby fine-tuning the rhythmic activity of these pathways (Cho et al., 2012; Marciano et al., 2014). REV-ERBα/β double-knockouts (*Nr1d1/2* ^−/−^) display significantly altered circadian wheel-running behavior and lipid metabolism (Cho et al., 2012). RORα KOs (RORα^sg/sg^) exhibit dyslipidemia, aberrant feeding and locomotion, atherosclerosis, hyper-inflammation, muscle atrophy and ataxia (Steinmayr et al., 1998). RORα is abundantly expressed in skeletal muscle where it directly interacts with p300 and MYOD to promote myogenesis and differentiation of muscle cells (Lau et al., 1999). These effects could at least partially explain the reduced skeletal muscle strength and increased atrophy observed in RORα^sg/sg^ mice (Steinmayr et al., 1998).

Cholesterol and intermediates (7-dehydrocholesterol, cholesterol sulfate) are RORα agonists that induce RORE-dependent transcriptional activation of downstream genes involved in lipid metabolism, at least in part when co-activated by p300 and PGC-1α (Kallen et al., 2002; Lau et al., 2004; Kang et al., 2007). In line, repression of RORα in skeletal muscle cells results in attenuated expression of a host of genes involved in triglyceride hydrolysis and fatty acid uptake and oxidation, as well as cholesterol homeostasis (Lau et al., 2004). Further, in a dominant-negative RORα model (RORα1ΔDE), alterations in skeletal muscle insulin signaling and glucose tolerance through modulation of the AKT2-AMPK signaling pathway were reported (Raichur et al., 2010). Collectively, these observations imply that RORα serves as a metabolic sensor and regulates energy homeostasis in the muscle by shifting preferential fuel utilization towards fatty acid catabolism (Lau et al., 2004; Kang et al., 2007).

REV-ERBs regulate skeletal muscle oxidative capacity by modulating mitochondrial biogenesis and fatty acid oxidation (Amador et al., 2018). Loss of REV-ERBα deactivates the LKB1-AMPK-SIRT1-PGC-1α axis resulting in reduced mitochondrial content and increased organelle clearance, collectively impairing endurance capacity (Woldt et al., 2013). The expression of several mitophagy and autophagy genes is upregulated in skeletal muscle of REV-ERB-null mice, associated with reduced skeletal muscle mass and decreased fiber size (Mayeuf-Louchart et al., 2017). Inversely, muscle-specific overexpression or pharmacological activation of REV-ERBα inhibit autophagy and protein ubiquitination, rescue mitochondrial number and respiratory function, and thereby enhance exercise capacity (Woldt et al., 2013; Mayeuf-Louchart et al., 2017). REV-ERBα also regulates muscle fiber-type distribution and is selectively expressed in glycolytic type IIB and intermediate type IIA muscle fibers, with a shift towards oxidative type I fibers in REV-ERBα-null mice (Pircher et al., 2005). These studies indicate a physiological role of REV-ERBs in the modulation of muscle mass and exercise capacity.

## Conclusion: Circadian Control of Exercise Adaptations

Exercise and the molecular clock are tightly inter-connected and mutually entrain each other (Figure 2). For example, BMAL1, PER2, and CRY1 expression modulates exercise capacity *via* regulation of mitochondrial function and fuel-optimization through modulation of lipid and glucose metabolism (Gutierrez-Monreal et al., 2020). Exercise, in turn, affects the molecular clock and induces expression of clock proteins (Rovina et al., 2021), leading to phase-shifts in the expression and oscillation patterns of clock factors in skeletal muscle (Wolff and Esser, 2012; Kemler et al., 2020), in line with altered expression of CRY1, PER2, and BMAL1 in trained human muscle biopsies promoting phase shifts in circadian rhythmicity (Zambon et al., 2003). Most circadian muscle genes show peak expression in the middle of the active phase, attributed to higher metabolic demands and increased contractile activity (McCarthy et al., 2007). AMPK, PGC-1α, and HIF-1α are just some of the many proposed candidates that mediate the interplay between exercise, skeletal muscle function, and circadian rhythmicity (Liu et al., 2007; Peek et al., 2017; Gabriel and Zierath, 2019). Exercise increases cellular energy demands, e.g., as manifested in reduced levels of ATP and NADH, activating AMPK and SIRT1 pathways, which modulate the stability of the PER2-CRY1 complex (Lamia et al., 2009; Jordan et al., 2017). Exercise capacity is also improved through AMPK-mediated activation of PPARδ, which regulates lipid metabolism and energy uncoupling in skeletal muscle (Jordan et al., 2017). PGC-1α, activated by exercise in skeletal muscle, stimulates mitochondrial biogenesis in response to energy stress. PGC-1α influences muscle function and exercise capacity by affecting angiogenesis, fiber-type transition, and RORα/γ-mediated BMAL1-CLOCK expression (Handschin et al., 2007; Liu et al., 2007). Exercise also modulates HIF-1α activity in the muscle, with ensuing clock-HIF interactions that regulate oxidative metabolism and anaerobic glycolysis (Peek et al., 2017). In summary, exercise affects the molecular clock in the muscle through complex modulation of many interconnected signaling pathways, and vice versa, the phase of the clock impacts muscle metabolism and function (Figure 2). It however is unclear whether the clock components exert these effects in their capacity as clock genes, or *via* additional biological functions in the muscle cell. This mutual interaction raises the question of when the best time-of-day to exercise is, a topic which is of high relevance in maximizing the beneficial effects of training.

Circadian differences in peak performance for strength and endurance activities are reflected in the greater number of world records in early evening compared to early morning (Atkinson and Reilly, 1996; Lok et al., 2020; Kusumoto et al., 2021). According to these studies, skeletal muscle strength changes with time-of-day, peaking during the late afternoon (∼16:00–20:00 h) (Douglas et al., 2021). In line, 12 weeks of strength and endurance training in the evening led to improved physical performance, muscle hypertrophy, and serum hormone concentrations compared to morning exercise (Kuusmaa et al., 2016). Similarly, in metabolically compromised humans, training in the afternoon had greater benefits on metabolic parameters like peripheral insulin sensitivity, adipose tissue lipolysis, fasting plasma glucose levels, exercise performance and fat mass (Savikj et al., 2019; Mancilla et al., 2021). However, these circadian fluctuations in peak endurance and strength performance cannot be replicated in all human studies, alluding to a much more complicated and multifactorial relationship between circadian rhythmicity of central and peripheral clocks, environmental and lifestyle factors including sleep, nutrition, psychological aspects, ambient temperature and ultimately muscle function (Facer-Childs and Brandstaetter, 2015; Aoyama and Shibata, 2020). Individual variance in biological clocks and preference for either morning/evening can be used to classify circadian phenotypes into early/intermediate/late chronotypes (ECT/ICT/LCT) (Duglan and Lamia, 2019). For example, peak performance depends not only on time-of-day but also on chronotype and time since entrained waking, with ECTs performing better in early-afternoon and LCTs in late-evening (Facer-Childs and Brandstaetter, 2015). Regular training at a particular time-of-day can entrain performance leading to greater adaptations at that time (Kusumoto et al., 2021). For practical purposes, this would imply that athletes should match regular training hours with the time-of-day of required peak performance at competitions. Notably however, the time of peak performance and of optimal training effect might not necessarily coincide. For example, endurance capacity in mice is highest at the dark/light phase transition, when muscle and liver glycogen storage is maximized (Maier et al., 2022). However, the markedly different transcriptome, proteome and phosphoproteome in skeletal muscle at Zeitgeber time 12 implies strong activation of signaling pathways important for training adaptation, for example, the AMPK signaling axis, reflecting a “train-low” paradigm with an improved outcome despite reduced performance and exacerbated perception of effort (Hearris et al., 2018). Finally, at least in mice, a circadian shift in activity, e.g., induced by daytime running, has very small effects on the phase of the molecular clock in muscle or liver (Adamovich et al., 2021; Maier et al., 2022). Furthermore, in stark contrast to the liver clock exhibiting a complete 12-h shift, daytime eating does not affect the muscle clock, implying a greater robustness of the latter towards perturbations (Maier et al., 2022).

In summary, as outlined, the “time to train” is strongly individualized and multifactorial, and our knowledge of the intersection of the molecular clock, muscle function, exercise capacity, and training effect still is rudimentary. For practical purposes, other commitments, logistical and infrastructural aspects, psychological factors, adjusting to members of a group in team sports, recovery and rehabilitation could complicate precise and personalized chrono-exercise. Therefore, for most people, finding a time to train that fits within personal schedules and preference most likely supersedes circadian considerations.
